# Moderating effects of sex on the impact of diagnosis and amyloid positivity on verbal memory and hippocampal volume

**DOI:** 10.1186/s13195-017-0300-8

**Published:** 2017-09-12

**Authors:** Jessica Z. K. Caldwell, Jody-Lynn Berg, Jeffrey L. Cummings, Sarah J. Banks, for the Alzheimer’s Disease Neuroimaging Initiative

**Affiliations:** Cleveland Clinic Lou Ruvo Center for Brain Health, 888 West Bonneville Avenue, Las Vegas, NV 89106 USA

**Keywords:** Alzheimer’s disease, Amyloid, Mild cognitive impairment (MCI), PET, Volumetric magnetic resonance imaging (MRI), Memory

## Abstract

**Background:**

Alzheimer’s disease (AD) impacts men and women differently, but the effect of sex on predementia stages is unclear. The objective of this study was to examine whether sex moderates the impact of florbetapir positron emission tomography (PET) amyloid positivity (A^+^) on verbal learning and memory performance and hippocampal volume (HV) in normal cognition (NC) and early mild cognitive impairment (eMCI).

**Methods:**

Seven hundred forty-two participants with NC and participants with eMCI from the Alzheimer’s Disease Neuroimaging Initiative (second cohort [ADNI2] and Grand Opportunity Cohort [ADNI-GO]) were included. All had baseline florbetapir PET measured, and 526 had screening visit HV measured. Regression moderation models were used to examine whether A^+^ effects on Rey Auditory Verbal Learning Test learning and delayed recall and right and left HV (adjusted for total intracranial volume) were moderated by diagnosis and sex. Age, cognition at screening, education, and apolipoprotein E ε4 carrier status were controlled.

**Results:**

Women with A^+^, but not those with florbetapir PET amyloid negative (A-),eMCI showed poorer learning. For women with NC, there was no relationship of A^+^ with learning. In contrast, A^+^ men trended toward poorer learning regardless of diagnosis. A similar trend was found for verbal delayed recall: Women with A^+^, but not A-, eMCI trended toward reduced delayed recall; no effects were observed for women with NC or for men. Hippocampal analyses indicated that women with A^+^, but not those with A^−^, eMCI, trended toward smaller right HV; no significant A^+^ effects were observed for women with NC. Men showed similar, though nonsignificant, patterns of smaller right HV in A^+^ eMCI, but not in men with A^−^ eMCI or NC. No interactive effects of sex were noted for left HV.

**Conclusions:**

Women with NC showed verbal learning and memory scores robust to A^+^, and women with A^+^ eMCI lost this advantage. In contrast, A^+^ impacted men’s scores less significantly or not at all, and comparably across those with NC and eMCI. Sex marginally moderated the relationship of A^+^ and diagnosis with right HV, such that women with NC showed no A^+^ effect and women with A^+^ eMCI lost that advantage in neural integrity; the pattern in men was less clear. These findings show that women with A^+^ eMCI (i.e., prodromal AD) have differential neural and cognitive decline, which has implications for considering sex in early detection of AD and development of therapeutics.

## Background

Florbetapir positron emission tomography (PET) amyloid positivity (A^+^) is a biomarker for fibrillar amyloid associated with a high likelihood of progression to Alzheimer’s disease (AD) dementia [1]. β-Amyloid (Aβ) accumulation has been linked to brain atrophy [1–3] and cognitive decline in AD [1–7]. However, findings have been mixed regarding whether and how A^+^ relates to cognitive dysfunction or hippocampal volume (HV) across the spectrum of normal cognition (NC), early mild cognitive impairment (eMCI), and AD [4, 8–13].

Some inconsistencies in the extant literature relate to disease stage included and concurrent versus longitudinal assessment of outcomes. For example, on one hand, although A^+^ may be detectable in NC, it may not be meaningfully related to concurrent cognition until development of eMCI [2, 14]. On the other hand, A^+^ may be strongly linked to retrospective longitudinal decline in NC but less predictive of future decline as the disease progresses to eMCI [4]. Other potentially explanatory factors include differences in sample size, type of Aβ measurement (e.g., binary positivity versus continuous measures of Aβ load), hippocampal segmentation and correction methodology [15–17], and type of outcome measure employed (e.g., screening measures such as overall scores on the Mini Mental State Examination [MMSE] versus more specific memory measures).

Although traditionally treated as a confound, sex differences in the relationship of A^+^ to cognition and HV may also explain conflicting findings. Recent investigations have revealed sex effects on hippocampal atrophy in normal aging, eMCI, and AD, though one study showed this only before controlling for Aβ levels [18–20]. Researchers have also shown that women’s established verbal memory advantage over men [20–23] appears to function as a form of sex-specific cognitive reserve, affording women equal or better cognitive performance compared with men via compensation despite positive biomarkers for AD, including mild to moderate levels of hippocampal atrophy [24] or fluorodeoxyglucose (^18^F-FDG)-PET hypometabolism [25]. Mechanisms of sex effects on hippocampal atrophy and cognition remain unclear, but as recent reviews and studies suggest, the etiology may include a complex interaction of effects of sex hormones; genetics (e.g., apolipoprotein E ε4 [APOEε4] carrier status); and psychosocial (e.g., differences in stress or coping), demographic (e.g., education), and lifestyle (e.g., exercise, smoking, and alcohol use) factors [20, 26]. Whether sex-specific reserve in cognition is seen in the face of A^+^ remains unclear. It is also unknown whether sex-specific hippocampal reserve exists for women with A^+^.

We examined whether sex moderates the effect of A^+^ on verbal learning and memory and HV in individuals with NC and eMCI. We hypothesized that sex would moderate the relationship of A^+^ and diagnosis with cognition such that women with NC would show a memory-related advantage over men that persists despite A^+^ and that women with eMCI would lose that advantage. We further hypothesized that sex would moderate the relationship of A^+^ and diagnosis with HV such that women with women with A^+^ and NC would show a neural robustness in hippocampal integrity but that women with eMCI would lose that advantage.

## Methods

### Participants

The Alzheimer’s Disease Neuroimaging Initiative (ADNI) is a longitudinal, multisite AD biomarker study (www.adni-info.org). The present investigation includes participants enrolled in the Alzheimer’s Disease Neuroimaging Initiative second cohort (ADNI2) and the Alzheimer’s Disease Neuroimaging Initiative Grand Opportunity Cohort (ADNI-GO) who had amyloid PET imaging at baseline and cognitive testing at screening (*n* = 742). Of included participants, 526 had screening visit HVs that met University of California, San Francisco (UCSF), quality control standards (UCSF Freesurfer Methods Quality Control [http://adni.loni.usc.edu/]). Participants were NC ADNI2 participants (*n* = 285) and participants with eMCI (ADNI2, *n =* 329; ADNI-GO, *n* = 128). ADNI required participants with NC to have MMSE scores of 24–30, a Clinical Dementia Rating (CDR) of 0, and no memory complaints. ADNI defined early eMCI as including MMSE [27] scores of 24–30, CDR [28] of 0.5, CDR Memory box score of 0.5 or greater, objective memory loss as assessed by education-adjusted scores on the Wechsler Memory Scale Logical Memory II test (raw scores 9–11 for >16 years of education, 8–15 for 5–9 years of education, 0–7 for 3–6 years of education), subjective memory complaint, and not meeting criteria for dementia [29].

### Hippocampal image processing

Fully processed HV and total intracranial volume (TIV) numerical values were downloaded from ADNI, with methods described in the UCSF Freesurfer Methods Quality Control document (www.adni.loni.usc.edu). In brief, magnetic resonance imaging (MRI) scans were obtained at baseline according to a standardized protocol (http://adni.loni.usc.edu/methods/mri-analysis/mri-acquisition/). Nonaccelerated T1-weighted images (multiplanar reconstruction or inversion recovery-spoiled gradient recalled acquisition in steady state) in Neuroimaging Informatics Technology Initiative format were preprocessed by the Mayo Clinic (gradient warping, scaling, B1 correction, and N3 inhomogeneity correction). Freesurfer (version 5.1; documented and freely available for download online [http://surfer.nmr.mgh.harvard.edu/]) was employed for motion correction and averaging [30] of multiple volumetric T1-weighted images (when more than one was available), removal of nonbrain tissue using a hybrid watershed/surface deformation procedure [31], automated Talairach transformation, segmentation of the subcortical white matter and deep gray matter volumetric structures (including hippocampus) [32, 33], intensity normalization [34], tessellation of the gray matter-white matter boundary, automated topology correction [35, 36], and surface deformation following intensity gradients to optimally place the gray/white and gray/cerebrospinal fluid borders at the location where the greatest shift in intensity defines the transition to the other tissue class [37, 38]. Visual quality control assessment of images was performed at UCSF (*see* www.adni.loni.usc.edu).

In the present analyses, we employed HV that passed UCSF-established quality control thresholds. We examined left and right HVs rather than a mean volume across hemispheres. This was based on significant testing for effect of hemisphere conducted prior to primary analyses described in the Statistical methods section (i.e., analysis of variance with sex, A^+^, and their interaction as between-subjects factors and hemisphere as a within-subject factor, predicting left and right hemisphere volumes). The results revealed significant effects of hemisphere on volume in NC [*F*(4,179) = 12.30, *p* = 0.001] and eMCI [*F*(4,347) = 25.87, *p* < 0.001] as well as a significant sex-by-A^+^-by-hemisphere interaction in eMCI [*F*(4,347) = 4.12, *p* = 0.043]. We adjusted left and right HVs for TIV according to procedures set forth by Mormino and colleagues [39]. In brief, adjusted hippocampal volume (aHV) was calculated according to the formula [aHV = raw HV − β(TIV − mean TIV)]. Mean TIV values were defined separately for NC and eMCI; mean TIV for the appropriate diagnostic group was subtracted from each individual’s TIV. This value was multiplied by the regression coefficient (β) obtained from a regression of TIV predicting HV in the appropriate diagnostic group. Finally, we calculated aHV by subtracting this value from raw left and right HVs for each participant.

### Florbetapir PET image processing

We downloaded fully processed ^18^F-FDG-PET binary positivity/negativity values from ADNI, where full protocols are also described (www.adni.loni.usc.edu). Florbetapir synthesis, image acquisition, and processing are additionally described in prior publications [4, 40, 41]. In brief, amyloid PET images were acquired at a variety of sites (4 × 5-minute frames obtained 50–70 minutes postinjection). Images were realigned; averaged; resliced to 1.5-mm^3^ voxel size; smoothed to 8-mm FWHM; and coregistered to baseline native space structural MRI scans, which were segmented and parcellated with Freesurfer version 5.3.0 to define cortical gray matter regions of interest (frontal, anterior/posterior cingulate, lateral parietal, lateral temporal) [39, 40]. A^+^ was determined by extracting weighted cortical retention means (regional standardized uptake value [SUVr]) from these regions, calculating average SUVr, and dividing by the cerebellar SUVr as a reference [40, 41]. In the present analyses, we used binary A^+^, employing the recommended 1.11 SUVr ratio threshold for cross-sectional analyses [40, 41].

### Apolipoprotein E carrier status

We downloaded apolipoprotein E (APOE) genotype data fully processed from ADNI (adni.loni.usc.edu). A binary variable was created, coding all individuals as APOE ε4 carriers (heterozygotes, *n* = 251; homozygotes, *n* = 53) or noncarriers.

### Clinical and cognitive measures

Cognitive outcome measures consisted of total learning and delayed free recall performance scores on the Rey Auditory Verbal Learning Test (RAVLT) [42]. We calculated total RAVLT learning scores by adding the five learning trial scores. We included modified total performance score on the Montreal Cognitive Assessment (MoCA) [43] as a measure of baseline cognitive status. To create a MoCA score that did not include a measure of memory performance, points earned for delayed list word recall were excluded from the total score, resulting in a maximum score of 25.

### Statistical methods

All analyses were performed using IBM SPSS Statistics software (IBM, Armonk, NY, USA) and the PROCESS macro [44, 45]. Mann-Whitney *U* tests were performed to examine group differences in demographic control variables. Four separate moderation regression analyses were performed to examine whether sex and diagnosis moderated main effects of amyloid status on RAVLT learning and delayed free recall scores as well as left and right HVs. For all analyses, we treated A^+^ as an independent variable, diagnosis as a moderator, and sex as a secondary moderator. Modified MoCA scores, age at screening visit, education, and APOE ε4 carrier status were included as covariates. All continuous covariates were mean-centered; dichotomous covariates were zero-centered.

For each of the four moderation analyses, outlying and influential data points were defined as those that failed two of the following three thresholds: (1) Cook’s D [*D* > 4/(*n* − *k* − 1)], where *n* = number of participants in the analysis and *k* = number of predictors; (2) leverage as defined by (2*k* + 2)/*n*, where *n* = number of participants in the analysis and *k* = number of predictors; and/or (3) Mahalanobis value greater than the chi-square cutoff at *p* < 0.001 (*df* = 6). On the basis of these criteria, one participant was excluded for the RAVLT learning analysis, none were excluded for the RAVLT delay analysis, and two were excluded for each of the HV analyses. We carefully inspected data for all participants whose data failed a single threshold measure in order to ensure no operator error created outlying data points, as well as to ensure that data points did not appear to belong to a different population.

## Results

### Demographics

Of 742 participants, 48.4% were women, 344 were A^+^, 304 were APOE ε4 allele carriers, and 457 were diagnosed with eMCI. The average age of the sample was 71.59 years (SD 6.98) and ranged from 55 to 91 years. Additional demographics by diagnosis, sex, and Aβ status are displayed in Table 1.

**Table 1:** Sample characteristics by diagnosis, sex, and amyloid status

	Normal cognition				Early mild cognitive impairment			
	Female (*n* = 154)		Male (*n* = 131)		Female (*n* = 205)		Male (*n* = 252)	
	Florbetapir-negative	Florbetapir-positive	Florbetapir-negative	Florbetapir-positive	Florbetapir-negative	Florbetapir-positive	Florbetapir-negative	Florbetapir-positive
Demographics								
No. of subjects	91	63	100	31	93	112	114	138
Age, years	70.8 (5.6)	72.4 (5.0)	72.3 (6.2)	77.8 (5.8)	69.5 (8.3)	70.8 (7.1)	69.2 (7.4)	73.7 (6.4)
Education, years	16.5 (2.5)	15.5 (2.6)	17.2 (2.3)	17.6 (2.4)	15.8 (2.3)	15.6 (2.8)	16.8 (2.5)	16.5 (2.7)
White, %	92.3	93.7	89	87.1	90.3	92	92.1	96.4
Hispanic, %	8.8	4.8	3	0	6.5	3.6	3.5	2.2
Cognition								
Modified MoCA	23.5 (1.6)	22.3 (1.7)	23.4 (1.7)	23.0 (1.6)	22.6 (2.1)	21.8 (2.4)	22.6 (2.0)	21.9 (2.5)
RAVLT Total Learning	48.8 (8.9)	47.0 (8.3)	44.4 (11.5)	37.7 (8.8)	45.0 (11.6)	37.6 (10.7)	37.2 (9.9)	32.0 (8.9)
RAVLT Delayed Recall	8.9 (3.4)	7.8 (3.6)	7.0 (4.5)	5.2 (3.1)	7.4 (4.5)	4.3 (4.2)	5.0 (3.7)	3.5 (3.2)
Brain volume^a^								
No. of subjects	59	34	70	16	77	84	86	100
Left, raw, mm^3^	3640.458 (408.312)	3626.059 (451.953)	3891.829 (495.200)	3716.563 (338.556)	3530.481 (502.509)	3339.369 (516.286)	3724.186 (579.737)	3438.300 (541.483)
Right, raw, mm^3^	3727.322 (444.720)	3718.941 (450.135)	3991.514 (460.636)	3712.375 (348.348)	3631.662 (493.285)	3338.345 (552.385)	3822.256 (612.419)	3562.970 (500.952)
Total ICV, raw, mm^3^	1.41 × 10^6^ (102,176.7)	1.41 × 10^6^ (122,896.4)	1.57 × 10^6^ (135,998.0)	1.58 × 10^6^ (151,662.0)	1.42 × 10^6^ (121,471.7)	1.41 × 10^6^ (108,602.5)	1.58 × 10^6^ (127,438.3)	1.61 × 10^6^ (149,837.7)
APOE ε4 carrier status								
ε4 allele carriers, *n*	23	29	17	15	19	76	32	93

*Abbreviations: APOE* Apolipoprotein E, *ICV* Intracranial volume, *MoCA* Montreal Cognitive Assessment, *RAVLT* Rey Auditory Verbal Learning Test

Mean (SD) data are shown for continuous variables

^a^ For brain volume analyses, females with normal cognition (*n* = 93), males with normal cognition (*n* = 86), females with early mild cognitive impairment (*n* = 161), and males with early mild cognitive impairment (*n* = 186)

Mann-Whitney *U* test results showed that, for NC, men were significantly older (*p* = 0.01) and more educated (*p* < 0.001) than women. For eMCI, men were also significantly older (*p* = 0.02) and more educated (*p* < 0.001) than women. There were no sex differences in modified MoCA score or APOE ε4 carrier status for NC or eMCI.

Mann-Whitney *U* tests showed that, for NC, those with A^+^ were significantly older (*p* < 0.001), less educated (*p* = 0.03), and less frequently APOE ε4 carriers (*p* < 0.001) than those with florbetapir positron emission tomography amyloid negativity (A^−^). No differences based on A^+^ were observed in modified MoCA scores (*p* < 0.001) for those with NC. For eMCI, those with A^+^ were significantly older (*p* < 0.001), had lower modified MoCA scores (*p* = 0.001), and were more often APOE ε4 carriers (*p* < 0.001) than those with A^−^. No differences were observed for education.

### Sex moderation of amyloid status and diagnosis effects on verbal learning and free recall

**Fig. 1:**
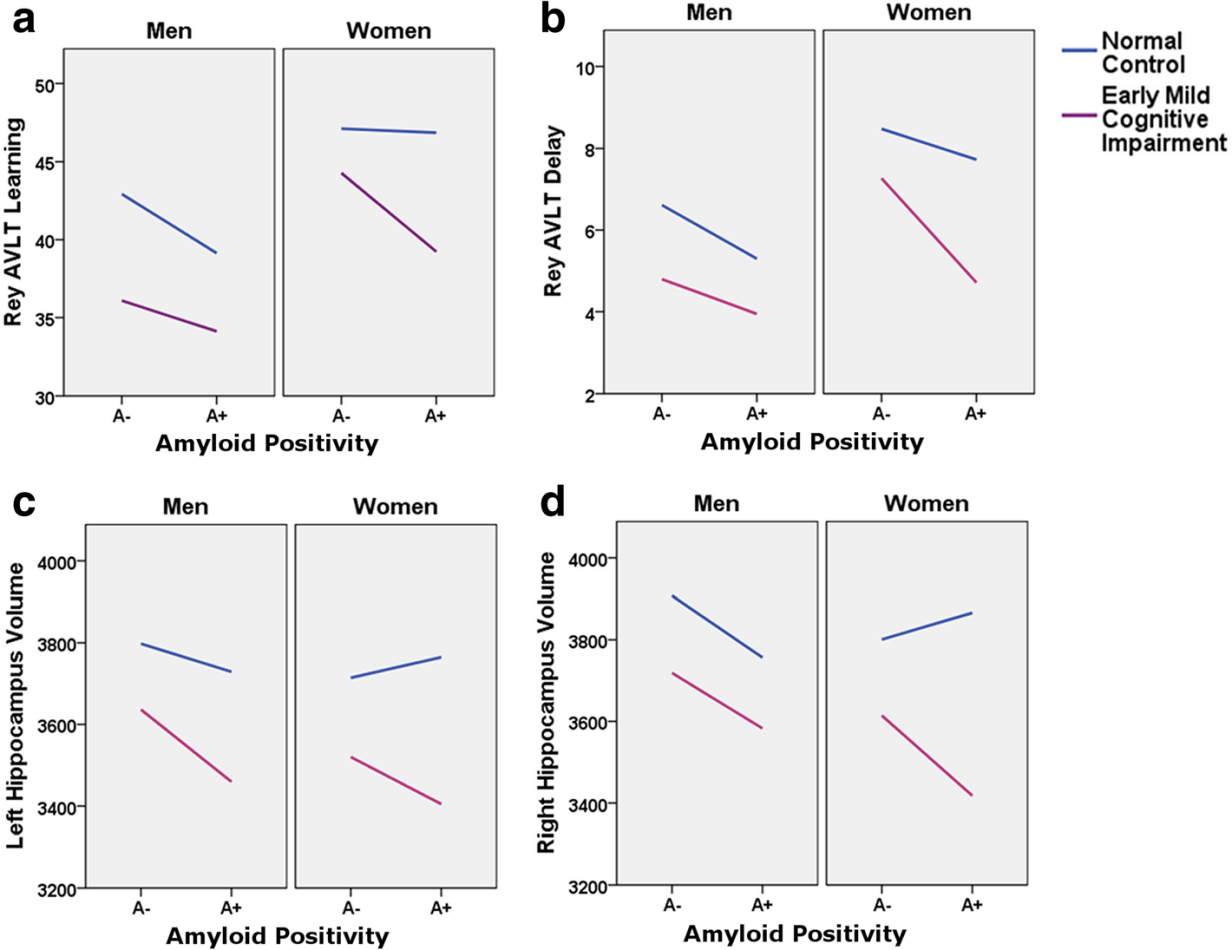
Sex moderation of diagnosis and amyloid status effects. Sex moderates effects of diagnosis and florbetapir positron emission tomography amyloid positivity (A^+^) on verbal learning (**a**) and marginally moderates effects on verbal delayed recall (**b**) and right hippocampal volume (HV; **d**), but it does not moderate effects on left HV (**c**). Specifically, learning and memory scores appear robust to A^+^ effects in women with normal cognition (NC; **a**, **b**). Women with prodromal AD (A^+^ early mild cognitive impairment [eMCI]) lose this advantage (**a**, **b**). In contrast, A^+^ impacts men’s verbal learning and memory scores comparably across NC and eMCI (**a**, **b**). Sex shows no moderating effect for left HV (**c**), but individuals of both sexes with eMCI show smaller left HV than individuals with NC. Sex marginally moderates the relationship of A^+^ and diagnosis with right HV, such that women with NC showed no effect of A^+^ on HV and women with prodromal AD lost that advantage in neural integrity (**d**). *A* ^*−*^ Florbetapir positron emission tomography amyloid negativity, *AVLT* Auditory Verbal Learning Test. Rey AVLT scores are group means. HV units are derived via correction for total intracranial volume

The overall model with A^+^, diagnosis, sex, and their interactions predicting verbal learning was significant [*F*(11,727) = 48.26, *p* < 0.001, *R* ^2^ = 0.39]. A three-way interaction showed that sex significantly moderated the effects of diagnosis and A^+^ on verbal learning [*t*(727) = −2.25, *p* = 0.02]. Parsing this interactive effect indicated that women were impacted differently by A^+^, depending on diagnosis. In particular, women with A^+^ eMCI, but not those with A^−^ eMCI, showed poorer learning [A^+^ eMCI, *t*(727) = −3.65, *p* < 0.01]. Similar A^+^ effects were not seen in women with NC [*t*(727) = −0.18, *p* = 0.85]. In contrast, A^+^ impacted men similarly regardless of diagnosis, with A^+^ showing trends toward poorer learning [NC, *t*(727) = −1.94, *p* = 0.05; eMCI, *t*(727) = −1.66, *p* < 0.01]. All findings were significant after controlling for age, education, modified MoCA score, and APOE ε4 carrier status. *See* Table 2 and Fig. 1a.

**Table 2:** Summary of regression moderation analyses for Rey Auditory Verbal Learning Test Verbal Learning and Free Recall memory test outcomes (*n* = 742)

	RAVLT Total Learning score^a^				RAVLT Delayed Free Recall score^b^			
	β Coefficient	*p* Value	95% CI		β Coefficient	*p* Value	95% CI	
Amyloid PET positivity	−2.766	0.001	−4.321	−1.21	−1.056	0.001	−1.689	−0.422
Diagnosis	−5.586	<0.0001	−7.122	−4.05	−1.791	<0.0001	−2.404	−1.178
Sex	6.308	<0.0001	4.804	7.812	1.854	<0.0001	1.245	2.463
A^+^ × diagnosis × sex	−6.616	0.025	−12.385	−0.847	−2.116	0.073	−4.431	0.199
A^+^ × diagnosis	−1.486	0.314	−4.382	1.411	−0.536	0.359	−1.683	0.611
A^+^ × sex	0.229	0.877	−2.677	3.135	−0.623	0.288	−1.773	0.528
Diagnosis × sex	0.689	0.639	−2.19	3.567	−0.562	0.341	−1.717	0.594
Age	−0.371	<0.0001	−0.473	−0.268	−0.095	<0.0001	−0.137	−0.053
Education	0.388	0.003	0.134	0.643	0.114	0.035	0.008	0.221
Modified MoCA	1.357	<0.0001	1.002	1.711	0.404	<0.0001	0.279	0.53
APOE ε4 carrier status	−1.713	0.0259	−3.219	−0.207	−0.817	0.008	−1.424	−0.211

*Abbreviations: A* ^*+*^ Florbetapir positron emission tomography amyloid positivity, *APOE* Apolipoprotein E, *MoCA* Montreal Cognitive Assessment, *PET* Positron emission tomography, *RAVLT* Rey Auditory Verbal Learning

Age, education, and modified MoCA score were centered at their means. Dichotomous variables were centered on zero (RAVLT total learning score overall model, *R* = 0.623, *p* < 0.0001; RAVLT Delayed Free Recall score overall model, *R* = 0.522, *p* < 0.0001)

^a^ *n* = 739 after exclusions

^b^ *n* = 741 after exclusions

The overall model with A^+^, diagnosis, sex, and their interactions predicting verbal delayed recall was also significant [*F*(11,729) = 32.94, *p* < 0.001, *R* ^2^ = 0.27]. Analyses showed trends toward a three-way interaction, suggesting that sex marginally moderated the effects of diagnosis and A^+^ on verbal delayed recall [*t*(729) = −1.18, *p* = 0.07]. Again, women with A^+^ eMCI, but not those with A^−^ eMCI, showed poorer delayed recall [*t*(729) = −3.64, *p* < 0.01], and no A^+^ effect was seen in women with NC [*t*(729) = −0.96, *p* = 0.34]. There was no effect of A^+^ on delayed recall for men with NC or eMCI [NC, *t*(729) = −1.36, *p* = 0.17; eMCI, *t*(729) = −1.08, *p* = 0.28]. Findings are controlled for age, education, modified MoCA score, and APOE ε4 carrier status. *See* Table 2 and Fig. 1b.

### Sex moderation of amyloid status and diagnosis effects on hippocampal volume

The overall model with A^+^, diagnosis, sex, and their interactions predicting left HV was significant [*F*(11,512) = 14.55, *p* < 0.001, *R* ^2^ = 0.25]. However, the three-way interaction of A^+^, diagnosis, and sex was not significant [*t*(512) = −0.35, *p* = 0.73], indicating no moderating effects of sex or diagnosis on A^+^ effects for the left HV. Main effects indicated that diagnosis related to smaller left HV across sexes, with men and women with eMCI showing smaller left HV than men and women with NC [*t*(512) = −5.88, *p* < 0.001]. Main effects of A^+^ on left HV showed a trend toward smaller left HV in men and women with A^+^ NC and eMCI [*t*(512) = −1.69, *p* = 0.09]. There was not a significant main effect of sex on left HV [*t*(512) = −1.30, *p* = 0.19]. *See* Table 3 and Fig. 1c.

**Table 3:** Summary of regression moderation analyses for left and right hippocampal volume outcomes (*n* = 526)

	Left hippocampus^a^				Right hippocampus^a^			
Variable	β Coefficient	*p* Value	95% CI		β Coefficient	*p* Value	95% CI	
Amyloid PET positivity	−77.566	0.092	−167.751	12.619	−104.569	0.021	−192.943	−16.195
Diagnosis	−246.297	<0.0001	−328.569	164.024	−249.308	<0.0001	−333.037	165.578
Sex	−54.713	0.193	−137.229	27.802	−66.609	0.111	−148.498	15.28
A^+^ × diagnosis × sex	−57.528	0.726	−380.114	265.058	−276.81	0.089	−596.41	42.789
A^+^ × diagnosis	−137.015	0.094	−297.34	23.309	−123.397	0.127	−281.968	35.173
A^+^ × sex	90.467	0.259	−66.718	247.652	77.968	0.329	−78.934	234.869
Diagnosis × sex	−61.618	0.454	−223.256	100.021	−135.762	0.097	−296.177	24.654
Age	−26.758	<0.0001	−32.976	20.539	−30.667	<0.0001	−36.752	−24.583
Education	0.312	0.969	−15.337	15.962	−8.534	0.281	−24.0581	6.989
Modified MoCA	34	0.001	13.599	54.4	31.479	0.003	10.901	52.057
APOE ε4 carrier status	−15.354	0.742	−107.004	76.297	−29.992	0.513	−120.083	60.099

*Abbreviations: A* ^*+*^ Florbetapir positron emission tomography amyloid positivity, *APOE* Apolipoprotein E, *MoCA* Montreal Cognitive Assessment, *PET* Positron emission tomography

Age, education, and modified MoCA scores were centered at their means. Dichotomous variables were centered on zero. Left hippocampus overall model (*R* = 0.495, *p* < 0.0001); right hippocampus overall model (*R* = 0.533, *p* < 0.0001)

^a^ *n* = 524 after exclusions

The overall model with A^+^, diagnosis, sex, and their interactions predicting right HV was significant [*F*(11,511) = 18.00, *p* < 0.001; *R* ^2^ = 0.28]. The three-way interaction of A^+^, diagnosis, and sex was a trend, suggesting that sex marginally moderated the effects of diagnosis and A^+^ on right HV [*t*(511) = −1.17, *p* = 0.09]. Parsing this interactive effect indicated that women were again impacted differently by A^+^ depending on diagnosis, with women with A^+^ eMCI, but not those with A^−^ eMCI, showing smaller right HV [*t*(511) = −2.71, *p* < 0.01]. There was no association of A^+^ with right HV in NC [*t*(511) = −0.77, *p* = 0.44]. For men, there was a trend toward A^+^ relating to smaller right HV in A^+^ eMCI and not A^−^ eMCI [*t*(511) = −1.75, *p* = 0.08]. No relationship was observed between A^+^ and right HV in NC men [*t*(511) = −1.58, *p* = 0.11]. *See* Table 3 and Fig. 1d.

## Discussion

In the present study, we examined the moderating effects of sex on the impact of diagnosis and A^+^ on verbal learning and memory and HV. The main finding was that sex moderated the effects of A^+^ and diagnosis on verbal learning. In addition, we showed that sex marginally moderated the effects of A^+^ and diagnosis on verbal delayed recall and that sex marginally moderated the effects of A^+^ and diagnosis on right HV. In contrast, no sex moderation effects were observed for left HV.

With respect to cognition, our findings specifically suggest that women’s advantage over men in verbal learning—and to a lesser extent delayed recall—was robust to A^+^ in NC. Moreover, in eMCI, only women with A^+^, and not those with A^−^, showed poorer learning—and to a lesser extent poorer delayed recall. These effects were observed after accounting for baseline cognitive status, age, education, and APOE ε4 carrier status. We conceptualize these findings as consistent with A^+^ eMCI representing a prodromal AD stage and A^−^ eMCI as representing suspected non-Alzheimer’s pathophysiology (SNAP). Our findings are consistent with literature showing better verbal memory performance in women [24] and positing a cognitive or memory reserve advantage for women with fewer prodromal AD traits (i.e., amnestic eMCI but moderate to large HV), but not with more prodromal AD traits (i.e., amnestic eMCI, dementia diagnosis, and small HV) [25, 26, 46]. Our findings are also partially consistent with a very recent study showing that women with low to moderate Aβ burden (but not high Aβ burden) had better verbal delayed recall than men and that this effect was specific to MCI versus NC or AD. A moderating effect of sex shown in the present study may help to explain some conflicting findings in the extant literature because, depending on sample size and diagnostic stage included, collapsing across sexes may lead to masked or exaggerated findings.

The present result showing that sex moderates the effect of A^+^ and diagnosis on learning and memory also has implications for clinical diagnosis of AD in women. Specifically, as has been suggested in the past [24, 25], memory reserve in women could delay prodromal AD diagnosis even in the face of positive biomarkers such as A^+^. However, the present results suggest that longitudinal assessment of the potentially steeper decline in memory for women between NC and prodromal AD, which is absent in SNAP, or combining measures of memory with other biomarkers, possibly with an approach that places heavier weight on biomarkers such as A^+^ early on, could increase diagnostic accuracy. This finding could also be relevant for development of therapeutics for AD, both with respect to inclusion criteria for trials (e.g., guidelines including a memory or learning score deficit requirement could exclude women with preclinical AD unintentionally) as well as outcome measures (e.g., the differing trajectories of memory decline in men and women could either exaggerate or mask important findings, depending on group composition, if sex is not considered).

With respect to HV, our present findings suggest moderating effects of sex for right HV. Similar to the pattern of results for the cognitive data, there was no relationship of A^+^ with right HV in women with NC. For women with eMCI, those with prodromal AD, but not SNAP, showed smaller right HV. The pattern in men was weaker and not significant, but it was similar. No moderating effects of sex were found for left HV.

Taken together, these findings may suggest that women have a neural reserve at the level of the hippocampus, such that hippocampal integrity is robust to effects of A^+^ in preclinical stages in women. Importantly, the present results do not imply that women have larger hippocampi and thus more volume to lose. Instead, they would suggest that neural reserve could be defined as a robustness to neurodegeneration, beginning at similar neural volume as men, when adjusting for TIV. Replication in even larger samples, as well as in samples of clinic-typical patients, will be important for understanding whether this is a true example of sex-specific neural reserve or whether findings would be significant in men with larger cohorts. If the latter were true, it might alternatively suggest that A^+^ is sensitive to concurrent HV loss in early clinical disease stages but not in NC. Larger cohort replication might also be helpful in determining whether the present lateralized findings might be consistent or whether bilateral effects would emerge. Certainly, left hemisphere effects might be expected, given the literature showing that women with NC and women with eMCI have stronger verbal memory [20, 24], and our lateralized results deserve further investigation.

Of note, in the present HV analysis, we intentionally employed a residual correction methodology for TIV [39], based on our specific sample composition as well as on guidelines recently published [17]. Previous work has suggested that a major source of variability in literature describing assessment of HV sex differences may be lack of [15], or differing methods for [16], correcting HV for total brain or intracranial volume. Use of a deliberate statistical approach taking sex into account at all levels may help to reduce or explain contradictory results in the literature relating A^+^ and sex to HV across NC and eMCI. Further research is needed to determine what pattern of sex moderation may exist at AD dementia stages at which women have been shown to have more rapid trajectories of decline [18, 46].

Strengths of the present study include use of a large, well-characterized study sample employing the prodromal AD diagnosis and rigorous control of potential confounding variables. Limitations include lack of longitudinal analysis, which could help to clarify causality, and use of a smaller cohort of individuals with HV data. It was also beyond the scope of the present analysis to explore ways in which HV may itself be a moderator of cognitive decline or to further probe HV at the level of subfields [47].

Future research is warranted on the longitudinal implications of these findings, as is replication in a larger cohort. In particular, because the present analysis employs clinically defined diagnostic stage groups in which men and women would be expected to express similar clinical symptoms, fine-grained examination of when exactly—or how much—pathology such as amyloid burden leads to cognitive, atrophic, and clinical symptom expression is needed. In addition, further validation and exploration of the currently used modification of the MoCA eliminating the memory component will also be important. Finally, examining the moderating effect of sex on other outcome measures, including hippocampal subfields, nonverbal memory, and resting state functional MRI, may be interesting.

## Conclusions

The present study shows that sex moderates the relationship of A^+^ and diagnosis with verbal learning performance and marginally moderates the effect of A^+^ and diagnosis on verbal delayed recall and right hemisphere HV. Whereas women with NC show learning and memory scores that are robust to A^+^ effects, women with prodromal AD lose this advantage; in contrast, A^+^ impacts men’s learning and memory scores in a less significant way or not at all and comparably across NC and eMCI. For right HV, the marginal sex moderation effect showed that women with NC had no effect of A^+^ on HV. Women with prodromal AD, but not those with SNAP, lost that advantage in right HV neural integrity; effects among men remain unclear. Further study of sex effects in prodromal AD and AD dementia has the potential to lead to clinical developments that increase diagnostic accuracy at early stages, as well as to increase the accuracy of treatment group formation and outcome assessment when developing novel therapeutics.

## Abbreviations

A^−^: Florbetapir positron emission tomography amyloid negativity
A^+^: Florbetapir positron emission tomography amyloid positivity
Aβ: β-Amyloid
AD: Alzheimer’s disease
ADNI: Alzheimer’s Disease Neuroimaging Initiative
ADNI2: Alzheimer’s Disease Neuroimaging Initiative Second Cohort
ADNI-GO: Alzheimer’s Disease Neuroimaging Initiative Grand Opportunity Cohort
aHV: Adjusted hippocampal volume
APOE: Apolipoprotein E
CDR: Clinical Dementia Rating
eMCI: Early mild cognitive impairment
^18^F-FDG: Fluorodeoxyglucose
HV: Hippocampal volume
ICV: Intracranial volume
MMSE: Mini Mental State Examination
MoCA: Montreal Cognitive Assessment
MRI: Magnetic resonance imaging
NC: Normal cognition
PET: Positron emission tomography
RAVLT: Rey Auditory Verbal Learning Test
SNAP: Suspected non-Alzheimer’s disease pathophysiology
SUVr: Regional standardized uptake value
TIV: Total intracranial volume
UCSF: University of California, San Francisco

## References

[1] Jack CR, Barrio JR, Kepe V (2013). Cerebral amyloid PET imaging in Alzheimer’s disease. Acta Neuropathol.

[2] Ewers M, Insel P, Jagust WJ, Shaw L, Aisen P, Petersen RC (2012). CSF biomarker and PIB-PET-derived β-amyloid signature predicts metabolic, gray matter, and cognitive changes in nondemented subjects. Cereb Cortex.

[3] Yau WY, Tudorascu DL, McDade EM, Ikonomovic S, James JA, Minhas D (2015). Longitudinal assessment of neuroimaging and clinical markers in autosomal dominant Alzheimer’s disease: a prospective cohort study. Lancet Neurol.

[4] Landau SM, Mintun MA, Joshi AD, Koeppe RA, Petersen RC, Aisen PS (2012). Amyloid deposition, hypometabolism, and longitudinal cognitive decline. Ann Neurol.

[5] Wang F, Gordon BA, Ryman DC, Ma S, Xiong C, Hassenstab J (2015). Cerebral amyloidosis associated with cognitive decline in autosomal dominant Alzheimer disease. Neurology.

[6] Ossenkoppele R, van Berckel BNM, Prins ND (2011). Amyloid imaging in prodromal Alzheimer’s disease. Alzheimers Res Ther.

[7] Jack CR, Lowe VJ, Weigand SD, Wiste HJ, Senjem ML, Knopman DS (2009). Serial PIB and MRI in normal, mild cognitive impairment and Alzheimer’s disease: implications for sequence of pathological events in Alzheimer’s disease. Brain.

[8] Apostolova LG, Hwang KS, Andrawis JP, Green AE, Babakchanian S, Morra JH (2010). 3D PIB and CSF biomarker associations with hippocampal atrophy in ADNI subjects. Neurobiol Aging.

[9] Bourgeat P, Chetelat G, Villemagne VL, Fripp J, Raniga P, Pike K (2010). β-Amyloid burden in the temporal neocortex is related to hippocampal atrophy in elderly subjects without dementia. Neurology.

[10] Chételat G, Villemagne VL, Bourgeat P, Pike KE, Jones G, Ames D (2010). Relationship between atrophy and β-amyloid deposition in Alzheimer disease. Ann Neurol.

[11] Frisoni GB, Lorenzi M, Caroli A, Kemppainen N, Nagren K, Rinne JO (2009). In vivo mapping of amyloid toxicity in Alzheimer disease. Neurology.

[12] Mattsson N, Tosun D, Insel PS, Simonson A, Jack CR, Beckett LA (2014). Association of brain amyloid-β with cerebral perfusion and structure in Alzheimer’s disease and mild cognitive impairment. Brain.

[13] Trzepacz PT, Hochstetler H, Yu P, Castelluccio P, Witte MM, Dell’Agnello G (2016). Relationship of hippocampal volume to amyloid burden across diagnostic stages of Alzheimer’s disease. Dement Geriatr Cogn Disord.

[14] Song Z, Insel PS, Buckley S, Yohannes S, Mezher A, Simonson A (2015). Brain amyloid-β burden is associated with disruption of intrinsic functional connectivity within the medial temporal lobe in cognitively normal elderly. J Neurosci.

[15] Tan A, Ma W, Vira A, Marwha D, Eliot L (2016). The human hippocampus is not sexually-dimorphic: meta-analysis of structural MRI volumes. Neuroimage.

[16] Perlaki G, Orsi G, Plozer E, Altbacker A, Darnai G, Nagy SA (2014). Are there any sex differences in the hippocampus volume after head-size correction? A volumetric and voxel-based morphometric study. Neurosci Lett.

[17] Nordenskjold R, Malmberg F, Larsson EM, Simmons A, Ahlstrom H, Johansson L (2005). Intracranial volume normalization methods: considerations when investigating gender differences in regional brain volume. Psychiatry Res Neuroimaging.

[18] Nosheny RL, Insel PS, Truran D, Schuff N, Jack CR, Aisen PS (2015). Variables associated with hippocampal atrophy rate in normal aging and mild cognitive impairment. Neurobiol Aging.

[19] Ardekani BA, Convit A, Bachman AH (2015). Analysis of the MIRIAD data shows sex differences in hippocampal atrophy progression. J Alzheimers Dis.

[20] Jack CR, Wiste HJ, Weigand SD, Knopman DS, Vemuri P, Mielke MM (2015). Age, sex, and APOE ε4 effects on memory, brain structure, and β-amyloid across the adult life span. JAMA Neurol.

[21] McCarrey AC, An Y, Kitner-Triolo MH, Ferrucci L, Resnick SM (2016). Sex differences in cognitive trajectories in clinically normal older adults. Psychol Aging.

[22] Murre JM, Janssen SM, Rouw R, Meeter M (2013). The rise and fall of immediate and delayed memory for verbal and visuospatial information from late childhood to late adulthood. Acta Psychol (Amst).

[23] Munro CA, Winicki JM, Schretlen DJ, Gower EW, Turano KA, Muñoz B (2012). Sex differences in cognition in healthy elderly individuals. Neuropsychol Dev Cogn B Aging Neuropsychol Cogn.

[24] Sundermann EE, Biegon A, Rubin LH, Lipton RB, Mowrey W, Landau S (2016). Better verbal memory in women than men in MCI despite similar levels of hippocampal atrophy. Neurology.

[25] Sundermann EE, Maki PM, Rubin LH, Lipton RB, Landau S, Biegon A (2016). Female advantage in verbal memory: Evidence of sex-specific cognitive reserve. Neurology.

[26] Mielke MM, Vemuri P, Rocca WA (2014). Clinical epidemiology of Alzheimer’s disease: assessing sex and gender differences. Clin Epidemiol.

[27] Folstein MF, Folstein SE, McHugh PR (1975). “Mini-mental state”: a practical method for grading the cognitive state of patients for the clinician. J Psychiatr Res.

[28] Morris JC (1993). Clinical Dementia Rating: current version and scoring rules. Neurology.

[29] Petersen RC, Aisen PS, Beckett LA, Donohue MC, Gamst AC, Harvey DJ (2010). Alzheimer’s Disease Neuroimaging Initiative (ADNI): clinical characterization. Neurology.

[30] Reuter M, Rosas HD, Fischl B (2010). Highly accurate inverse consistent registration: a robust approach. Neuroimage.

[31] Ségonne F, Dale AM, Busa E, Glessner M, Salat D, Hahn HK (2004). A hybrid approach to the skull stripping problem in MRI. Neuroimage.

[32] Fischl B, Salat DH, Busa E, Albert M, Dieterich M, Haselgrove C (2002). Whole brain segmentation: automated labeling of neuroanatomical structures in the human brain. Neuron.

[33] Fischl B, Salat DH, van der Kouwe AJ, Makris N, Ségonne F, Quinn BT (2004). Sequence-independent segmentation of magnetic resonance images. Neuroimage.

[34] Sled JG, Zijdenbos AP, Evans AC (1998). A nonparametric method for automatic correction of intensity nonuniformity in MRI data. IEEE Trans Med Imaging.

[35] Fischl B, Liu A, Dale AM (2001). Automated manifold surgery: constructing geometrically accurate and topologically correct models of the human cerebral cortex. IEEE Trans Med Imaging.

[36] Segonne F, Pacheco J, Fischl B (2007). Geometrically accurate topology-correction of cortical surfaces using nonseparating loops. IEEE Trans Med Imaging.

[37] Dale AM, Fischl B, Sereno MI (1999). Cortical surface-based analysis. I. Segmentation and surface reconstruction. Neuroimage.

[38] Dale AM, Sereno MI (1993). Improved localization of cortical activity by combining EEG and MEG with MRI cortical surface reconstruction: a linear approach. J Cogn Neurosci.

[39] Mormino EC, Betensky RA, Hedden T, Schultz AP, Amariglio RE, Rentz DM (2014). Synergistic effect of β-amyloid and neurodegeneration on cognitive decline in clinically normal individuals. JAMA Neurol.

[40] Landau SM, Breault C, Joshi AD, Pontecorvo M, Mathis CA, Jagust WJ (2013). Amyloid-β imaging with Pittsburgh compound B and florbetapir: comparing radiotracers and quantification methods. J Nucl Med.

[41] Landau SM, Marks SM, Mormino EC, Rabinovici GD, Oh H, O’Neil JP (2012). Association of lifetime cognitive engagement and low β-amyloid deposition. Arch Neurol.

[42] Rey A (1941). L’examen psychologique dans les cas d’encéphalopathie traumatique. Arch Psychol (Geneve).

[43] Nasreddine ZS, Phillips NA, Bédirian V, Charbonneau S, Whitehead V, Collin I (2005). The Montreal Cognitive Assessment, MoCA: a brief screening tool for mild cognitive impairment. J Am Geriatr Soc.

[44] IBM (2015). IBM SPSS Statistics for Windows.

[45] Hayes AF (2013). Introduction to mediation, moderation, and conditional process analysis: a regression based approach.

[46] Koran MEI, Wagener M, Hohman TJ, Alzheimer’s Neuroimaging Initiative (2017). Sex differences in the association between AD biomarkers and cognitive decline. Brain Imaging Behav.

[47] Gale SD, Baxter L, Thompson J (2016). Greater memory impairment in dementing females than males, relative to sex-matched healthy controls. J Clin Exp Neuropsychol.

